# A self-regenerable soot sensor with a proton-conductive thin electrolyte and a nanostructured platinum sensing electrode

**DOI:** 10.1038/srep37463

**Published:** 2016-11-18

**Authors:** Peiling Lv, Takenori Ito, Akihide Oogushi, Kensaku Nakashima, Masahiro Nagao, Takashi Hibino

**Affiliations:** 1 Graduate School of Environmental Studies, Nagoya University, Nagoya 464-8601, Japan; 2 Graduate School of Information Science, Nagoya University, Nagoya 464-8601, Japan; 3 Engine Component Department, Isuzu Motors Limited, Kanagawa 252-0881, Japan; 4 R&D Operation, Development Division, IBIDEN co., Ltd, Gifu 501-0695, Japan

## Abstract

In recent years, exhaust sensors have become increasingly attractive for use in energy and environmental technologies. Important issues regarding practical applications of these sensors, especially for soot measurements, include the further development of ion-conductive electrolytes and active electrode catalysts for meeting performance and durability requirements. Herein, we design a proton conductor with a high breakdown voltage and a sensing electrode with high sensitivity to electrochemical carbon oxidation, enabling continuous soot monitoring with self-regeneration of the sensor. A Si_0.97_Al_0.03_H_x_P_2_O_7-δ_ layer with an excellent balance between proton conductivity and voltage endurance was grown on the surface of a Si_0.97_Al_0.03_O_2-δ_ substrate by reacting it with liquid H_3_PO_4_ at 600 °C. Specific reactivity of the electrochemically formed active oxygen toward soot was accomplished by adding a Pt-impregnated Sn_0.9_In_0.1_H_x_P_2_O_7-δ_ catalyst into a Pt sensing electrode. To make the best use of these optimized materials, a unipolar electrochemical device was fabricated by configuring the sensing and counter electrodes on the same surface of the electrolyte layer. The resulting amperometric mode sensor successfully produced a current signal that corresponded to the quantity of soot.

Soot emitted from combustion engines is having an increasing impact on public health and the environment^1^, which has resulted in stricter restrictions on soot emissions worldwide^2^. Currently, emitted soot is removed by trapping the particles on ceramic filters, after which the filters are burned^3^,^4^. In order to better establish this removal system, the automobile and manufacturing industries require innovative soot detecting technologies. In urban environments, optical methods are used for monitoring the soot concentration^5^,^6^,^7^; however, they require complicated and expensive equipment. Several simpler potentiometric and amperometric soot sensors that employ solid electrolytes (yttria-stabilized zirconia^8^,^9^ and SnP_2_O_7_^10^,^11^,^12^,^13^), an electrical insulator (alumina)^14^,^15^,^16^, or a semiconductor (GaN)^17^ have been proposed. One such sensor, which consists of proton-conducting Sn_0.9_In_0.1_H_x_P_2_O_7-δ_ or SnP_2_O_7_-SnO_2_ solid electrolyte, produces active oxygen at a Pt sensing electrode via the anodic water vapor oxidation reaction. This oxygen species shows catalytic activity for carbon oxidation at temperatures of 50 °C or higher:


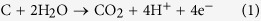


Reaction (1) generates a change in polarization resistance corresponding to a change in the soot concentration at the sensing electrode. In addition, this reaction self-regenerates the sensing electrode, which enables the soot concentration in the sample gas to be monitored in real time, unlike the case for other sensing devices.

At present, there are still two requirements that have not yet been met for using such a sensor. One is that the solid electrolyte must be able to withstand a bias voltage of at least 3 V, because Reaction (1) can proceed only under such polarization conditions. Unfortunately, the SnP_2_O_7_-based solid electrolytes are not able to meet this requirement under long-term use due to the electrochemical reduction of Sn^4+^ to metallic Sn (*E*_*0*_ = −0.13 V *vs*. SHE)^18^, which causes a short-circuit between the two electrodes. Various members of the MP_2_O_7_ (M = Ce, Ti, Zr, and Si) family have also been employed^19^,^20^,^21^,^22^,^23^,^24^,^25^, among which SiP_2_O_7_ possesses a very high redox potential of −2.40 V *vs*. SHE^26^. However, the proton conductivity reported for this material is one or two orders of magnitude lower than that of Sn_0.9_In_0.1_H_x_P_2_O_7-δ_, which is likely due to the low proton concentration in the bulk as well as the large grain-boundary resistance^27^. The second requirement is that the sensing electrode provides a higher sensitivity toward soot with a faster response speed, as compared with the Pt electrode. The weak soot sensing properties of currently used electrodes are primarily due to the poor kinetics of the platinum black catalyst used in Reaction (1), which in turn results from the limited number of reaction sites available for soot oxidation on the catalyst surface.

In this study, we investigate two approaches to meet these requirements. First, we impart high proton conductivity to the surface of a sintered Si_1-x_Al_x_O_2-δ_ substrate. By reacting the substrate with liquid H_3_PO_4_ at elevated temperatures, a thin, dense Si_1-x_Al_x_H_x_P_2_O_7-δ_ film forms on the substrate, which provides the required proton conductivity. We next increase the number of reaction sites for soot oxidation by impregnating the surface of the Sn_0.9_In_0.1_H_x_P_2_O_7-δ_ powder with Pt clusters, and then add this catalyst to the sensing electrode. The validity of these approaches is examined as a means of improving a soot sensor operating at a bias voltage of 3 V and at a temperature of 150 °C.

## Results

### Formation and identification of Si_0.97_Al_0.03_H_x_P_2_O_7-δ_ layers

The crystal structure of Al-doped SiO_2_ synthesized at 1400 °C was determined by X-ray diffraction (XRD) to be primarily cristobalite (Figure S1). For an aluminum content of 3 mol%, the lattice constants increased from {a, b, c} = {4.973, 4.973, 6.918} to {a, b, c} = {4.975, 4.975, 6.937}; however, for an aluminum content of 5 mol%, there was no additional change in the lattice constants, and a second Al_2_O_3_ phase was observed. Thus, we conclude that the maximum solid solubility of Al for this system is 3 mol%. This Al solubility is far lower than those shown in the known Al_2_O_3_-SiO_2_ phase diagram^28^, where the solution state reaches equilibrium. However, the sintering temperature for the Al-doped SiO_2_ substrate was 1400 °C, which is lower than that typically used for Al_2_O_3_-SiO_2_ systems. Thus, the Al_2_O_3_-SiO_2_ system in the substrate remains in a non-equilibrium steady state, causing the low solubility limit of the Al in the silica.

**Figure 1:**
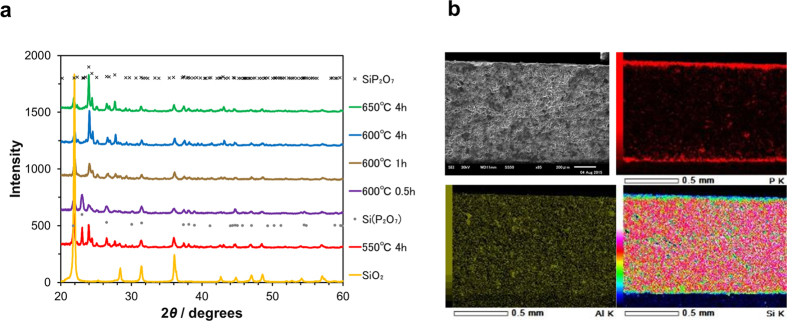
Characterization of Si_1-x_Al_x_H_x_P_2_O_7-δ_ layer. (**a**) XRD patterns for Si_0.97_Al_0.03_O_2-δ_ and Si_1-x_Al_x_H_x_P_2_O_7-δ_ films and (**b**) SEM/EDX micrographs of a cross section of Si_0.97_Al_0.03_H_x_P_2_O_7-δ_/Si_0.97_Al_0.03_O_2-δ_ composite.

The crystal structure of the surface of a Si_0.97_Al_0.03_O_2-δ_ substrate before and after treatment with phosphoric acid between 550 and 650 °C was measured via XRD (Fig. 1a). At 550 °C, a mixed crystal structure including phases of SiO_2_ (tetragonal, space group: P41212), SiP_2_O_7_ (monoclinic, space group: P21/n), and Si(P_2_O_7_) (hexagonal, space group: P63) was observed. At 600 °C, the diffraction intensity corresponding to the SiO_2_ and Si(P_2_O_7_) phases was lower, while the intensity associated with the SiP_2_O_7_ phase was higher. At 650 °C, only the SiO_2_ and SiP_2_O_7_ phases were present. Varying the duration of the thermal processing at 600 °C yielded the following results: thermal processing for 0.5 hours gave a blend of the SiO_2_, SiP_2_O_7_, and Si(P_2_O_7_) phases; at 1 hour, the Si(P_2_O_7_) phase disappeared; at 4 hours, the diffraction intensity associated with the SiP_2_O_7_ phase increased, while that associated with the SiO_2_ phase weakened; at 6 hours, no further changes were observed in the diffraction intensity associated with either phase.

To obtain additional information on the compounds, we performed scanning electron microscopy (SEM) and energy dispersive X-ray spectrometry (EDX) measurements on a cross section of the substrate after 4 hours of thermal processing at 600 °C (Fig. 1b). From the obtained elemental mappings for P, Si, and Al, we determined that an approximately 40-μm-thick layer was formed in a laminar configuration on the surface, and that the elements were uniformly distributed throughout the layer. The Al/(Si + Al) molar ratio for the layer agreed with the composition of the source materials within the experimental error (Figure S2). A noteworthy feature of this compound layer is that it is fine-grained to an extent similar to that for the Si_0.97_Al_0.03_O_2-δ_ substrate (Figure S3). Based on these results, the Si_0.97_Al_0.03_H_x_P_2_O_7-δ_ layer forms on the substrate surface according to the following reaction:


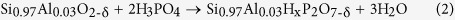


In the experiments discussed below, substrates subjected to thermal processing for 4 hours at 600 °C in phosphoric acid were used as electrolyte samples.

### Conductivity of Si_0.97_Al_0.03_H_x_P_2_O_7-δ_ layers fabricated on Si_0.97_Al_0.03_O_2-δ_ substrates

**Figure 2:**
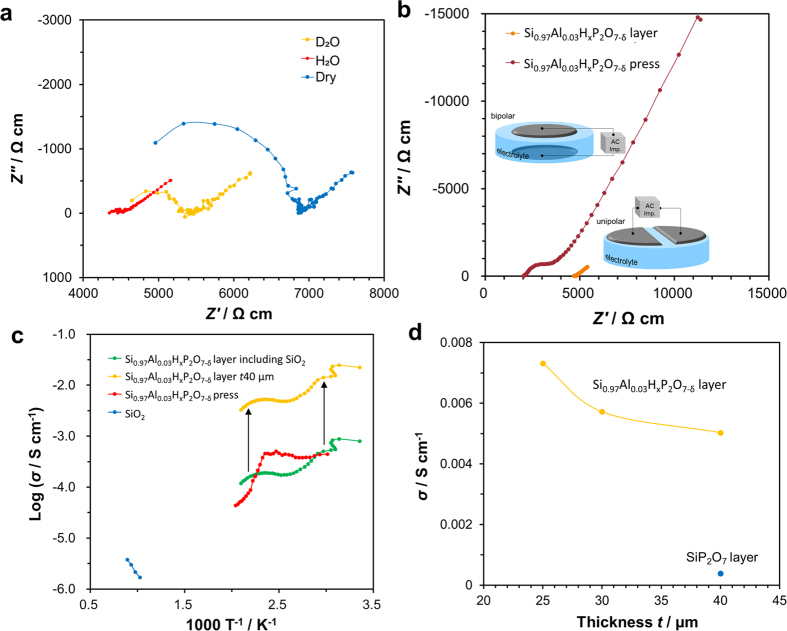
Electrochemical properties of Si_0.97_Al_0.03_H_x_P_2_O_7-δ_ layer and pressed pellet at 150 °C. (**a**) Impedance spectra of Si_0.97_Al_0.03_H_x_P_2_O_7-δ_/Si_0.97_Al_0.03_O_2-δ_ composite disk under non-humidified and humidified conditions, (**b**) impedance spectra of Si_0.97_Al_0.03_H_x_P_2_O_7-δ_/Si_0.97_Al_0.03_O_2-δ_ composite measured in unipolar mode and Si_0.97_Al_0.03_H_x_P_2_O_7-δ_ pressed pellet measured in bipolar mode, (**c**) electrical conductivities of Si_0.97_Al_0.03_H_x_P_2_O_7-δ_/Si_0.97_Al_0.03_O_2-δ_ composite and pressed pellet, Si_0.97_Al_0.03_H_x_P_2_O_7-δ_ layer, and Si_0.97_Al_0.03_O_2-δ_ substrate, and (**d**) electrical conductivity of Si_0.97_Al_0.03_H_x_P_2_O_7-δ_ layer as a function of thickness. For comparison, data for a 40-μm-thick SiP_2_O_7_ layer are also included.

The Si_0.97_Al_0.03_H_x_P_2_O_7-δ_ synthesized in this study was a thin, fine-grained phosphate layer on the Si_0.97_Al_0.03_O_2-δ_ substrate. To characterize the electrical conductivity of the layers, we arranged two electrodes in a unipolar configuration. First, to evaluate the effect of the electrode configuration on the conductivity, we used both unipolar and bipolar electrodes to measure the conductivity of a slab of yttria-stabilized zirconia (YSZ). Both methods yielded equivalent values, demonstrating that the unipolar configuration poses no problems (Figure S4). To investigate the proton conductivity in the Si_0.97_Al_0.03_H_x_P_2_O_7-δ_ layers, we measured variations in the impedance at 150 °C under conditions of no added H_2_O, added H_2_O, and added D_2_O (Fig. 2a). The ohmic resistance was lower when H_2_O was added than when no H_2_O was added, indicating an increased concentration of protons, which are the carriers of electric current in this system. Moreover, adding D_2_O yielded an ohmic resistance larger than that observed for H_2_O addition; this H/D isotope effect confirms that the charge carriers are protons. Partially substituting Si^4+^ cations with lower valency Al^3+^ cations gives rise to positively-charged defects [Reaction (3)], which react with water vapor through Reaction (4) or (5) to introduce hydrogen bonds in the form of 2OH_o_^∙^ or (HP_2_O_7_)_P2O7_^∙^:


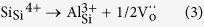



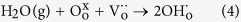



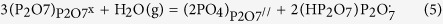


We attribute this to the fact that the protons are involved in electrical conduction^29^. In the above reactions, 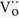, 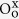, 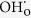 (HP_2_O_7_)P_2_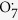 respectively denote oxygen vacancies, lattice oxide ions, and the two types of interstitial protons. Consequently, in what follows, we will refer to a layer of Si_0.97_Al_0.03_H_x_P_2_O_7-δ_ formed on a Si_0.97_Al_0.03_O_-δ_ substrate as a “Si_0.97_Al_0.03_H_x_P_2_O_7-δ_/Si_0.97_Al_0.03_O_2-δ_ composite”.

Figure 2b compares the impedance of the composite to that of a pure pressed pellet of Si_0.97_Al_0.03_H_x_P_2_O_7-δ_ at 150 °C. The bulk resistance of the composite is approximately twice that of the pressed pellet because it is primarily composed of insulating SiO_2_. Indeed, conductivity measurements of an unprocessed Si_0.97_Al_0.03_O_2-δ_ substrate yielded a value on the order of 10^−6 ^S cm^−1^ at 800 °C, but the electrical resistance was too large to allow for measurements in the range of 100–200 °C (Fig. 2c). In contrast, the impedance component associated with the grain-boundary resistance of the composite was dramatically smaller than that of the pressed pellet. We measured the temperature dependence of the proton conductivities obtained from the bulk resistance and the grain-boundary resistance of both samples (Fig. 2c). The result shown here for the composite body was obtained from Scheme S1 using the conductivity of the Si_0.97_Al_0.03_H_x_P_2_O_7-δ_ layer alone and assuming a thickness *a* of 40 μm. In contrast to the conductivity of the composite, the conductivity of the Si_0.97_Al_0.03_H_x_P_2_O_7-δ_ layer alone was on the order of 10^−3 ^S cm^−1^ (0.0053 S cm^−1^ at 150 °C), which is more than an order of magnitude greater than that of the pressed pellet. This high conductivity is due to the fact that the Si_0.97_Al_0.03_H_x_P_2_O_7-δ_ layer was grown while maintaining the high crystallinity of the substrate, which yielded a highly fine-grained substance with reduced grain-boundary resistance. On the other hand, the conductivity of all three samples was observed to decrease with temperature at or above 100 °C. This behavior may be explained by noting that temperature increases under conditions of low relative humidity cause the protons dissolved in a solid to be expunged to the exterior of the solid. However, measurements of the temperature dependence of the conductivity during the second temperature-raising process yielded values equivalent to those of the first temperature-raising process (Figure S5). This indicates that protons, even if expunged from the interior of the solid at high temperatures, re-enter the solid at low temperatures. We believe that Reactions (4) and (5) most likely proceed reversibly at the temperatures studied here. An interesting phenomenon that emerges from our observations is that the proton conductivity of the Si_0.97_Al_0.03_H_x_P_2_O_7-δ_ layer at 150 °C increases as the layer thickness decreases (Fig. 2d). A similar phenomenon was observed for BaCe_0.8_Y_0.2_O_3-α_ surfaces and was explained by noting that the proton conductor becomes increasingly surface-rich as its thickness decreases, resulting in an increase in the relative volume of proton injection into the interior of the solid^30^. Note that the data in the figure correspond to values of the proton conductivity of SiP_2_O_7_ layers fabricated on non-doped silica substrates. Without the contribution of protons provided by Reaction (4), only the protons produced by Reaction (5) are present, resulting in low conductivity. Based on these observations, we conclude that, although the conductivity of Si_0.97_Al_0.03_H_x_P_2_O_7-δ_/Si_0.97_Al_0.03_O_2-δ_ composite conducting structures is lower than those reported for samples and composites involving the Sn_0.9_In_0.1_H_x_P_2_O_7-δ_ system, these structures are well suited for use as electrolytes in sensing applications requiring high voltage tolerance and high mechanical strength.

### Characterization and electrochemical studies of sensing electrode materials in bipolar cell mode

**Figure 3:**
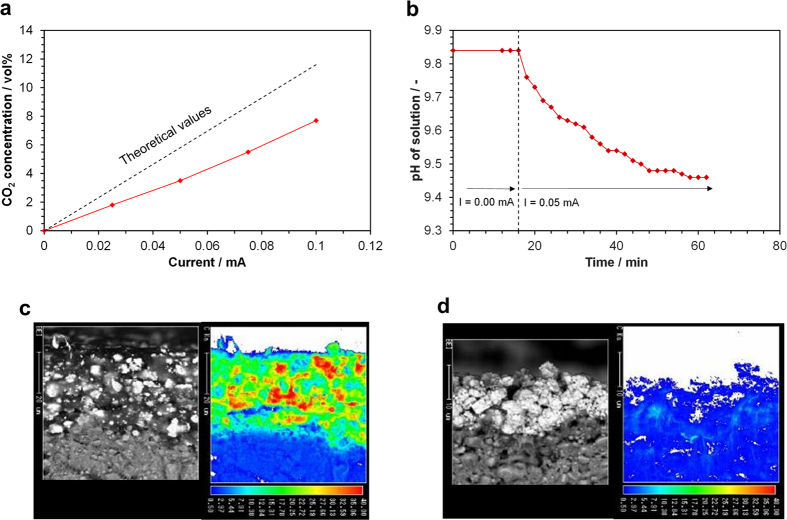
Electrode properties of a Pt/Sn_0.9_In_0.1_H_x_P_2_O_7-δ_ sensing electrode pre-mixed with soot measured at 150 °C. (**a**) CO_2_ concentration as a function of current and (**b**) transient change in pH of a NaOH solution bubbled with the outlet gas from the electrode chamber. SEM/EPMA micrographs of a cross section taken at the interface of the electrolyte and sensing electrode (**c**) before and (**d**) after applying the current.

The electrochemical soot oxidation represented in Reaction (1) was inspected using a bipolar cell with a Pt + Sn_0.9_In_0.1_H_x_P_2_O_7-δ_ + soot mixed electrode, where the Sn_0.9_In_0.1_H_x_P_2_O_7-δ_ powder was not impregnated with Pt. It should also be noted that the soot was not flowed into the electrode chamber, but instead added directly into the electrode to prevent contamination of the gas chromatograph (GC) column by the soot. The electrochemical cell was galvanostatically polarized, and the concentration of CO_2_ formed by anodic polarization of the electrode in the outlet gas was compared with the theoretical value calculated based on Reaction (1) using Faraday’s law (Fig. 3(a)). No product was observed at the open-circuit voltage, while CO_2_ and a small amount of O_2_ were the major oxidation products under anodic polarization. The CO_2_ concentration was lower than the theoretical value at each current, giving a current efficiency (actual CO_2_ concentration divided by the theoretical value) of approximately 0.59. To further assess the characteristics of electrochemical soot oxidation over the Pt + Sn_0.9_In_0.1_H_x_P_2_O_7-δ_ + soot mixed electrode, SEM and electron probe microanalysis (EPMA) observations at the electrolyte/electrode interface were carried out before and after applying the voltage. The voltage was applied such that the current was a constant 0.05 mA, and the outlet gas from the electrode chamber was bubbled through a NaOH solution with an initial pH of 9.84. The pH decreased continuously until reaching about 9.44, after which the experiment was stopped (Fig. 3(b)). The EPMA elemental mappings showed that the soot particles distributed over the electrode before the polarization nearly disappeared after the anodic polarization (Fig. 3(c) and (d)) due to the formation of a homogeneous network of Sn_0.9_In_0.1_H_x_P_2_O_7-δ_ ionomers within the electrode. These results strongly suggest that the sensing electrode can self-regenerate without the accumulation of deposited soot when the soot is supplied as floating matter to the electrode chamber.

**Figure 4:**
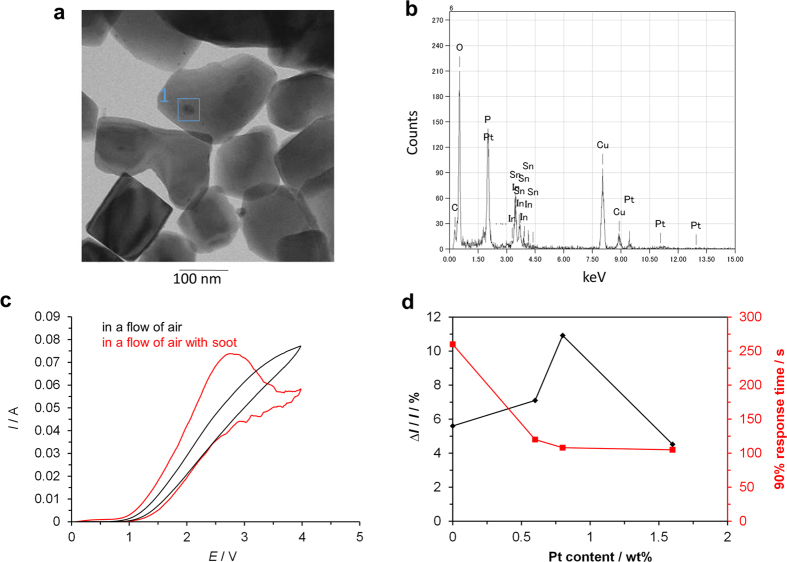
Microstructural, compositional, and electrochemical analyses of Pt + Pt/Sn_0.9_In_0.1_H_x_P_2_O_7-δ_ sensing electrodes. (**a**) TEM image and (**b**) EDX spectrum of the catalyst with a Pt content of 0.8 wt%. (**c**) *In situ* CVs in flowing sample gas streams with and without soot at a scan rate of 10 mV s^−1^, and (**d**) sensitivity toward soot (∆*I*/*I*_*0*_) and 90% response time as a function of Pt content in the catalyst.

The sensitivity and response of the present sensor were based on the specific reactivity of active oxygen toward soot, which is further ensured by enhancing the catalytic activity of the sensing electrode for this reaction. We thus attempted to increase the number of reaction sites by impregnating the Sn_0.9_In_0.1_H_x_P_2_O_7-δ_ ionomer surface with Pt metal (hereafter denoted Pt/Sn_0.9_In_0.1_H_x_P_2_O_7-δ_). The Pt content was adjusted to be less than 2 wt% because of the small Brunauer–Emmett–Teller (BET) specific surface area of the ionomer (7.4 m^2 ^g^−1^). Transmission electron microscopy (TEM) imaging of the catalyst revealed that the Pt cluster size distribution was in the range of several nm to ~20 nm (Fig. 4(a)). In addition, the EDX spectrum of the Pt cluster shown in Fig. 4(a) revealed that the atomic percentage of platinum (Pt:Sn:P:O = 2:11:23:64 at.%) was approximately ten times higher than that in the corresponding source material (Fig. 4(b)), which reflects the large particle size of the analyzed Pt cluster.

Prior to the performance tests, to determine the applied voltage necessary for electrochemical soot oxidation, *in situ* cyclic voltammetry (CV) measurements were conducted using the bipolar cell with a Pt + Pt/Sn_0.9_In_0.1_H_x_P_2_O_7-δ_ mixed electrode. In the present case, the sensing electrode was exposed to flowing sample gas streams with and without soot (3.930 mg m^−3^). The current started to flow at around 1 V regardless of the presence or absence of soot (Fig. 4(c)), indicating that the water vapor oxidation reaction occurs at the sensing electrode. The current increased almost linearly with the voltage until the terminal voltage in the absence of soot, while a large anodic peak current attributable to the carbon oxidation (discussed in the next section) at around 2.8 V was observed in the presence of soot. From these results, the applied voltage necessary for soot sensing operation was determined to be 3 V. The Pt content in the Pt/Sn_0.9_In_0.1_H_x_P_2_O_7-δ_ catalyst is important for determining the number of reaction sites for soot oxidation. Optimization of the Pt content was evaluated by applying a constant voltage of 3 V to the bipolar cell with Pt + Pt/Sn_0.9_In_0.1_H_x_P_2_O_7-δ_ mixed electrodes (Pt content of 0–1.6 wt%), followed by measuring the change in current upon turning the soot on and off (∆*I*) as well as the 90% response time for the soot. The sensitivity toward soot, which corresponds to the ratio of ∆*I* to the base current *I*_*0*_, increased with the Pt content and reached a maximum value at 0.8 wt% (Fig. 4(d)). In addition, the 90% response time was found to be minimized at 0.8 wt% or higher, which was therefore determined to be the optimal Pt content for the sensing electrode.

### Performance tests of a sensor constructed from optimized components in unipolar cell mode

**Figure 5:**
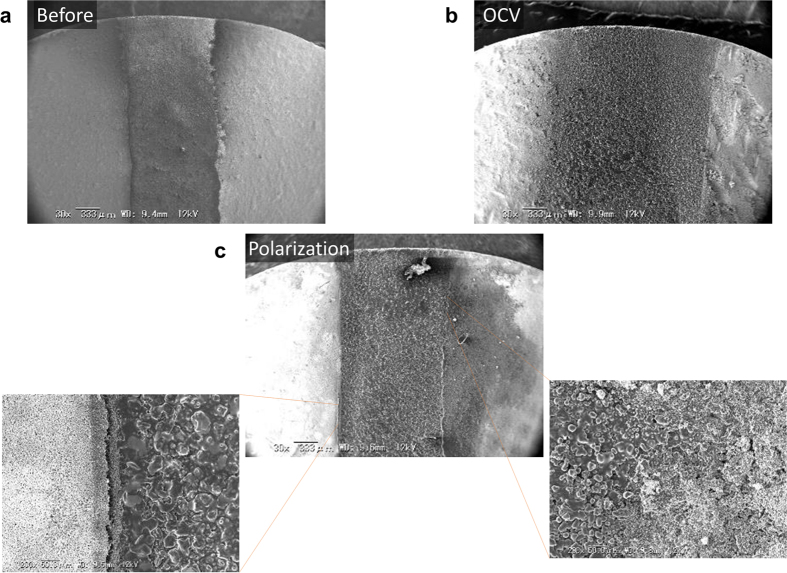
Top-view SEM images of the gap between the sensing and counter electrodes for a unipolar sensor acquired (**a**) before tests, after exposure to soot under (**b**) open-circuit and (**c**) anodic polarization conditions. Enlarged views of the sensing and counter electrode sides are also shown.

A sensor device was fabricated using the Si_0.97_Al_0.03_H_x_P_2_O_7-δ_/Si_0.97_Al_0.03_O_2-δ_ electrolyte with the Pt + Pt/Sn_0.9_In_0.1_H_x_P_2_O_7-δ_ mixed sensing and counter electrodes in the unipolar cell configuration. The microstructure of the zone around the gap between the two electrodes was analyzed by SEM. Based on a comparison of the SEM images before and after exposure to a flowing soot stream under open-circuit conditions, the soot was markedly deposited on the device surface, so that it protruded from the gap to the inner part of the electrode (Fig. 5(a) and (b)). This behavior is similar to that for other resistive-type soot sensors^14^,^15^,^16^,^17^ and caused short-circuiting of the sensor. Indeed, the open circuit voltage (OCV) for the present device decreased from around 10 mV to almost zero when exposed to the soot (Figure S6). However, when the soot was supplied under anodic polarization, the sensing electrode was unchanged from that prior to exposure to the soot, while the counter electrode was covered with the soot (Fig. 5(c)). As emphasized by the corresponding enlarged views, protrusion of the soot was prevented at the edge of the sensing electrode, in contrast to the counter electrode. The OCV of the device was also confirmed to return almost to its initial value after the measurement. The above effect is recognized as a self-regeneration ability that is beneficial for the continuous monitoring of soot. Based on these results, an applied voltage of 3 V was used in the subsequent experiments.

**Figure 6:**
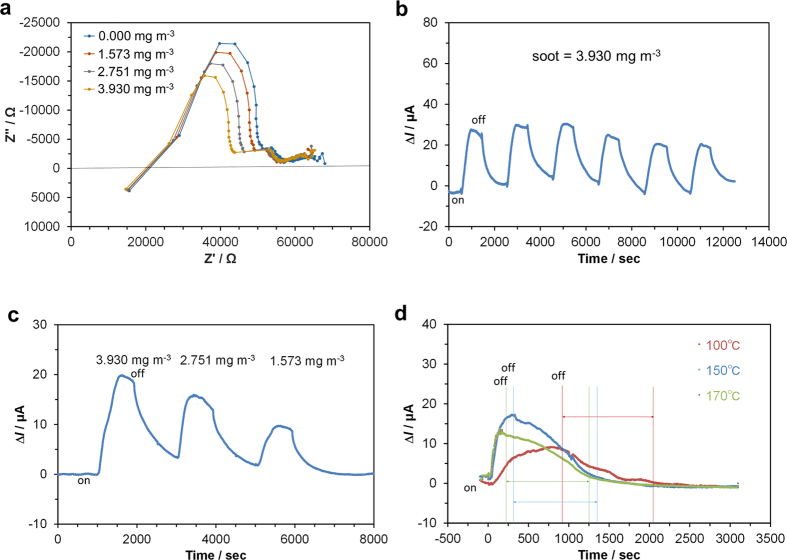
Sensing performance of the unipolar sensor at 150 °C. (**a**) Impedance spectra at a bias voltage of 3 V, and current responses upon repeatedly turning on and off the soot supply at (**b**) a constant soot concentration of 3.930 mg m^−3^, (**c**) various soot concentrations, and (**d**) various temperatures.

AC impedance measurements on the device were performed in flowing sample gas streams with various soot concentrations. Nyquist plots for the device showed that the activation polarization resistance decreased with increasing soot concentration (Fig. 6(a)). This is because Reaction (1) is more kinetically favorable for the anode reaction compared to the water vapor oxidation reaction, which will be demonstrated in the next section. On the other hand, the intercept of the impedance line and the real axis at high frequency, which corresponds to the ohmic resistance, was not dependent on the soot concentration, which is different to the result obtained for other types of soot sensors^14^,^15^,^16^,^17^. Similarly, the considerably distorted arc at low frequency, which is attributed to the diffusion resistance, was scarcely influenced by the soot concentration.

To evaluate the potential of this device for soot monitoring, the sensing properties were tested repeatedly (1–6 times) by supplying the sample gas with a soot concentration of 3.930 mg m^−3^, giving an ∆*I* of approximately 25 μA in each case (Fig. 6(b)). The correlation between the soot concentration and the current signal was also evaluated, showing a quantitative change in ∆*I* with the soot concentration (Fig. 6(c)). For each soot concentration, the sensitivity (∆*I*/*I*_*0*_) is not in agreement with the ratio based on the total resistance, including the ohmic resistance, activation polarization resistance, and diffusion resistance; however, it is in agreement with the ratio estimated from the sum of the ohmic resistance and activation polarization resistance. This is probably due to uncertainty in the diffusion resistance obtained at a high bias voltage of 3 V. Therefore, we conclude that the present device is promising for the real-time monitoring of soot. However, the recovery speed of the sensor was slower than its startup speed, particularly at high soot concentrations. This problem could not be simply ascribed to the kinetics because the recovery time was not sensitive to the operating temperature of the sensor (Fig. 6(d)). A possible external factor for this problem is the slow release of soot deposited on the wall of the sample gas line, as evidenced by the change in color of the gas line from transparent to brown during the sensing tests.

## Discussion

The ignition temperature for soot over platinum black in atmospheric air was at least 350 °C (Figure S7), whereas the electrochemical oxidation of the soot proceeded at 150 °C (Fig. 3). This is attributable to the formation of an active oxygen species via the water vapor oxidation reaction. An accelerating effect of Pt on Reaction (1) has also been reported in the literature^31^,^32^,^33^. We now discuss the reaction kinetics for such electrochemical soot oxidation on the Pt catalyst. The CV profile in the presence of soot (Fig. 6(c)) shows a small anodic band between 2.5 and 3.5 V in the reverse scan, in addition to the large anodic peak in the forward scan. The appearance of the small band can be interpreted as follows. A Pt-OH species, which is assumed to be active in oxidizing carbon, is formed at around 2.8 V in the forward scan. Further anodic polarization oxidizes Pt-OH to a PtO species that is assumed to be inactive with respect to the carbon oxidation, and thus the anodic current drastically decreases at high potentials. On the other hand, the PtO species is reduced to the Pt-OH species in the reverse scan, which again gives rise to an anodic current due to the carbon oxidation. Therefore, it is suggested that Reaction (1) comprises the following two steps:


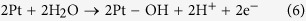



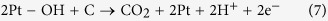


A similar mechanism for hydrocarbon oxidation was proposed by Brummer *et al*.^34^ and Heo *et al*.^35^. Evidence for this mechanism is provided by Raman spectra measured in the range of 100–2000 cm^−1^ for various applied voltages through a Pt + carbon mixed electrode (Figure S8). A band appeared at 918 cm^−1^ when a voltage was applied, and is assigned to the Pt-OH bending mode^36^. It is thus reasonable to conclude that the active oxygen species for carbon oxidation is OH^−^ combined with Pt, which is the origin of both the soot sensing and self-regeneration properties of the sensing electrode of the present sensor.

## Conclusions

We have developed a new amperometric sensor for the continuous monitoring of soot concentration based on the formation of active oxygen species that react with soot. Reaction of a Si_0.97_Al_0.03_O_2-δ_ substrate with liquid H_3_PO_4_ at 600 °C lead to the growth of a robust and dense Si_0.97_Al_0.03_H_x_P_2_O_7-δ_ layer that exhibited significantly low grain-boundary resistance for proton transport. By impregnating the Sn_0.9_In_0.1_H_x_P_2_O_7-δ_ particles with nanosized Pt clusters, the sensitivity and responsivity of the sensing electrode to soot were effectively improved, owing to an increased number of reaction sites for electrochemical soot oxidation. When a bias voltage of 3 V was applied to the sensor with a unipolar electrode configuration at 150 °C, the current signal responded to changes in the soot concentration without short-circuiting between the electrodes due to the accumulation of deposited soot.

## Methods

### Materials

#### Synthesis of Si_0.97_Al_0.03_H_x_P_2_O_7-δ_ layers and Si_0.97_Al_0.03_H_x_P_2_O_7-δ_ powders

Si_0.97_Al_0.03_H_x_P_2_O_7-δ_ layers were fabricated on the surfaces of sintered Si_0.97_Al_0.03_O_2-δ_ substrates as follows. Aluminum nitrate enneahydrate (Wako Pure Chemical Industries, 0.58 g) was dissolved in 150 ml of water, amorphous silica (Wako Pure Chemical Industries, 3.0 g) was added, and the mixture was heated while stirring at 200 °C until the water evaporated. The resulting powder was molded and pre-sintered in air for 4 hours at 500 °C. The product was ground in a mortar and re-molded using a uniaxial press into a pellet, which was then heated to 1400 °C in air for 10 hours to yield a sintered Si_0.97_Al_0.03_O_2-δ_ substrate. This sintered substrate was polished to a thickness of 1 mm using water-resistant polishing paper and was then cleaned in an ultrasonic bath. The resulting silica slab (0.25 g) was immersed for 1 hour in an 85% phosphoric acid solution (10 g), after which it was subjected to thermal processing at 600 °C in air for 4 hours. The phosphorylated silica slab was cleaned for 20 minutes with distilled water to eliminate any residual phosphoric acid.

Si_0.97_Al_0.03_H_x_P_2_O_7-δ_ powder was synthesized using previously reported methods^19^. The Si_0.97_Al_0.03_H_x_P_2_O_7-δ_ powder obtained as described above was added to 85% phosphoric acid to yield a mole fraction of 1:2.2, and was then heated while stirring at 150 °C until the water evaporated. The slurry thus obtained was transferred to a quartz beaker and subjected to thermal processing at 600 °C for 4 hours in air. Using this compound, we formed two samples: (1) a pressed pellet for conductivity measurements formed from the powder using a uniaxial press (2.6 MPa cm^−2^), and (2) a film for characterizing the properties of sensing electrodes formed by blending 1.00 g of the powder with 0.04 g of PTFE powder in a mortar and cold rolling the resulting mixture to a thickness of 250 μm on a laboratory rolling mill.

#### Synthesis of electrodes

Pt/Sn_0.9_In_0.1_H_x_P_2_O_7-δ_ powders were synthesized according to the procedure described in ref. 37. Sn_0.9_In_0.1_H_x_P_2_O_7-δ_ power (0.075 g) was dispersed in 120 ml of water, chloroplatinic acid 6 hydrate (0.0214 g) was added, and the mixture was heated while stirring at 150 °C until the water evaporated. The powder thus obtained was transferred to an alumina boat, where it was subjected to thermal processing for 1 hour at 200 °C in a 60 ml/min argon flow. To the resulting Pt/Sn_0.9_In_0.1_H_x_P_2_O_7-δ_ powder (0.035 g) was added 0.15 g of Pt paste (No. 8105 Tokuriki Honten), and the mixture was blended in a mortar. The resulting product was applied as a coating to the electrolyte and dried at 120 °C to obtain an electrode.

#### Characterization

The crystal phases of the Si_0.97_Al_0.03_H_x_P_2_O_7-δ_ powder were identified by XRD (MiniflexII, Rigaku). Diffraction patterns were obtained using Cu Kα rays (λ = 1.5432 Å) at a tube voltage of 45 kV and a tube current of 20 mA. Observations of the morphology of the electrolyte, analysis of its composition, and surface observations of the sensor elements were conducted at an applied voltage of 30 kV via SEM (VE-8800, Keyence) and EDX (JSM-6610A, JEOL). The soot ignition temperature was measured via TG-DTA (DTG-60, Shimadzu). Pt powder was added to soot (standard soot powder, Isuzu Motors Ltd.) to yield a 5.76 wt% Pt mixed powder. This powder was transferred to an alumina pan and heated from room temperature to 580 °C at a rate of 5 °C/min in air.

The resistance of the electrolyte was measured via the AC impedance method using a Solatron 1260 analyzer and a Solatron 1287 interface. Measurements were made over the frequency range of 0.1 to 10^6^ Hz at a voltage amplitude of 10 mV. The ionic conductivity was computed from the values of the ohmic resistance and grain-boundary resistance. Equations (8) and (9) were used to compute the conductivity for same-side (unipolar) and front-and-back-side (bipolar) configurations as follows (Scheme S1):


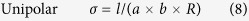



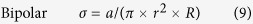


where *r* is the electrode radius, *a* is the thickness of the conductive layer, *b* is the length of the electrodes, *l* is the distance between the electrodes, and *R* is the resistance of the electrolyte. Humidified air at room temperature (*P*_*H*_*2*_*O*_ = 0.03 atm) was supplied at 60 mL min^−1^ to allow conductivity measurements to proceed in a humid environment. To measure the H/D isotope effect on the electrical conductivity, light water was replaced with heavy water in the humidifying gas supply.

The carbon composition of the Pt + Sn_0.9_In_0.1_H_x_P_2_O_7-δ_ sensing electrode pre-mixed with soot was determined before and after the anodic polarization using electron probe microanalysis (EPMA: EPMA-1610, Shimadzu) coupled with SEM. The dispersion state of the Pt clusters in the Pt/Sn_0.9_In_0.1_H_x_P_2_O_7-δ_ catalyst was analyzed using transmission electron microscopy (TEM: JEM2100F, JEOL) in conjunction with EDX (JED-2300T JEOL) spectroscopy.

#### Two types of electrochemical cells

Two different procedures and apparatuses were employed to measure various electrochemical characteristics: (1) a bipolar cell comprising two electrode chambers, where soot was pre-mixed into the sensing electrode or was continuously supplied from a lab-made soot generator to the sensing electrode, and (2) a unipolar cell set up in a bench test apparatus, where soot was supplied in a similar manner. The physically mixed Si_0.97_Al_0.03_H_x_P_2_O_7-δ_-PTFE composite membrane was employed as the electrolyte to optimize the sensing electrode in procedure (1), while the sintered Si_0.97_Al_0.03_H_x_P_2_O_7-δ_/Si_0.97_Al_0.03_O_2-δ_ composite disk was examined as the electrolyte for soot sensing in procedure (2).

A standard soot powder (Isuzu Motors Ltd.) was supplied as the soot in procedure (1), while soot was also supplied by burning a commercial incense stick in a plastic container, followed by dilution with water vapor-saturated air at a flow rate of 200 mL min^−1^, in both procedures (1) and (2). The sample gas was cooled using circulated water to room temperature during the experiments. The PM2.5 concentration in the sample gas was measured using a handheld PM2.5 monitoring device (CW-HAT200 ChinaWay).

In the bipolar cell, the sensing electrodes were prepared by mixing Sn_0.9_In_0.1_H_x_P_2_O_7-δ_ or Pt/Sn_0.9_In_0.1_H_x_P_2_O_7-δ_ powder (17 mg) in the presence (7 mg) or absence of soot powder with a Pt paste (75 mg) in the mortar. The counter electrode consisted of only Pt paste. The sensing and counter electrodes (area: 0.5 cm^2^) were painted on opposite faces of the electrolyte and then baked at 120 °C for 4 hours in atmospheric air. Electrochemical soot oxidation was investigated as follows. Argon saturated with water vapor was supplied to the soot pre-mixing sensing electrode at a flow rate of 30 mL min^−1^ under open-circuit or galvanostatic anodic polarization conditions. The CO_2_, CO, and O_2_ concentrations in the outlet gas from the electrode were measured at each current using an online gas chromatograph (CP-4900 Varian). CV and sensing measurements of the Pt + Pt/Sn_0.9_In_0.1_H_x_P_2_O_7-δ_ mixed electrodes were conducted using the two-electrode method. The sensing electrodes were supplied with the soot-containing sample gas and the counter electrode was exposed to atmospheric air. Voltammograms in the presence or absence of soot were obtained at a scan rate of 10 mV s^−1^. Sensing tests were performed by applying a voltage of 3 V to the cell. The current through the cell was recorded as the sensor signal. All of the experiments were carried out at a temperature of 150 °C.

In the unipolar cell configuration, the sensing electrode consisted of Pt and Pt/Sn_0.9_In_0.1_H_x_P_2_O_7-δ_, where the Pt content in the Pt/Sn_0.9_In_0.1_H_x_P_2_O_7-δ_ catalyst was determined to be 0.8 wt% based on the results of the above experiments. Several modifications to the experimental conditions and procedures were made for the bipolar experiments. Specifically, the counter electrode was identical to the sensing electrode, one side of the electrolyte, arranging the two electrodes, was continuously supplied with water vapor-saturated air with various soot concentrations (0–3.930 mg m^3^), and the gap between the two electrodes was 1 mm. The sensor device and apparatuses employed in this experiment are illustrated in Scheme S2. The impedance spectra of the device were acquired at a bias voltage of 3 V in the frequency range of 0.1–10^6 ^Hz with an AC amplitude of 20 mV. Unless otherwise stated, the other sensing tests were the same as those described above.

## Additional Information

**How to cite this article**: Lv, P. *et al*. A self-regenerable soot sensor with a proton-conductive thin electrolyte and a nanostructured platinum sensing electrode. *Sci. Rep.* **6**, 37463; doi: 10.1038/srep37463 (2016).

**Publisher’s note:** Springer Nature remains neutral with regard to jurisdictional claims in published maps and institutional affiliations.

## Supplementary Material

Supplementary Information
