# Supplementary material for: A self-regenerable soot sensor with a proton-conductive thin electrolyte and a nanostructured platinum sensing electrode
Source: Sci Rep. 2016 Nov 18;6:37463. doi: 10.1038/srep37463 (PMC5114587; doi:10.1038/srep37463)

Supplementary Information

A self-regenerable soot sensor with a proton-conductive thin electrolyte and a nanostructured platinum sensing electrode

Peiling Lv[1], Takenori Ito[2], Akihide Oogushi[3], Kensaku Nakashima[4], Masahiro Nagao[1] and Takashi Hibino[1*]

[1]Graduate School of Environmental Studies, Nagoya University, Nagoya 464-8601, Japan

[2]Graduate School of Information Science, Nagoya University, Nagoya 464-8601, Japan

[3]Engine Component Department, Isuzu Motors Limited, Kanagawa 252-0881, Japan

[4]R&D Operation, Development Division, IBIDEN co., Ltd, Gifu 501-0695, Japan

[*]hibino@urban.env.nagoya-u.ac.jp

[+]These authors contributed equally to this work.

Figure S1. XRD patterns for undoped SiO_2 and Al-doped SiO_2 sintered disks.

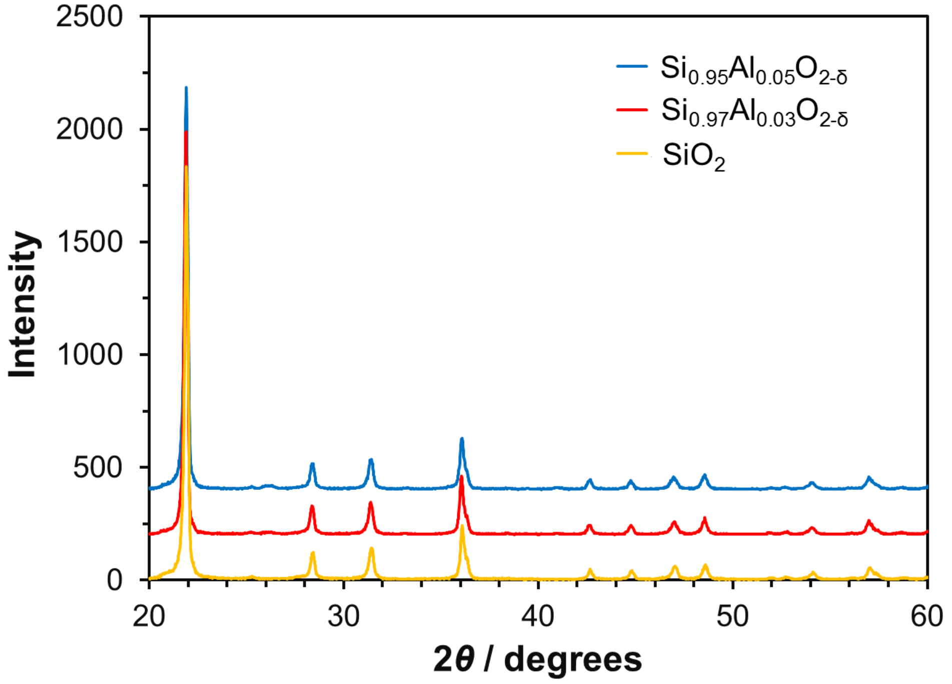

Figure S2. Al/(Si + Al) molar ratio obtained by EDX analysis.

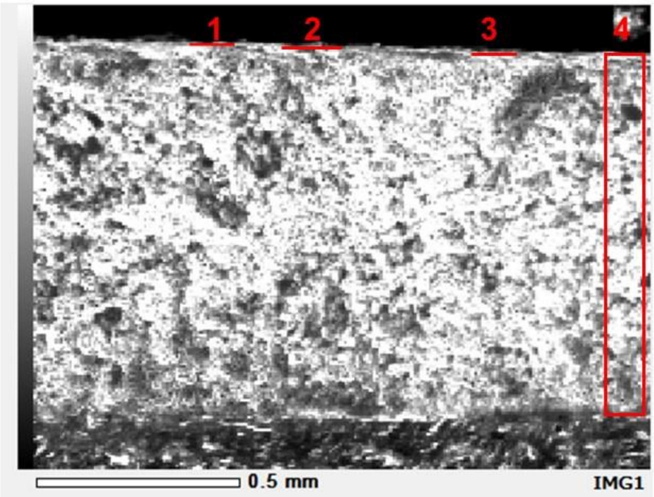

	Al/(Si + Al) molar ratio by EDX analysis
1	0.030
2	0.031
3	0.031
4	0.030

Figure S3. Cross-sectional SEM images taken at the interface of the $Si_{0.97}Al_{0.03}H_xP_2O_{7-\delta}$ layer and $Si_{0.97}Al_{0.03}O_{2-\delta}$ substrate.

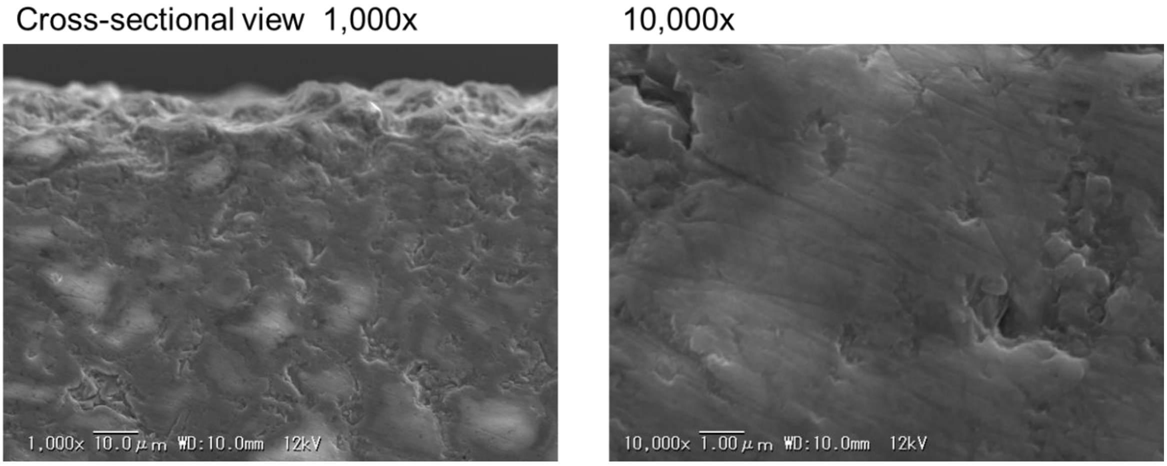

Figure S4. Temperature dependence of conductivity for YSZ measured in unipolar and bipolar modes.

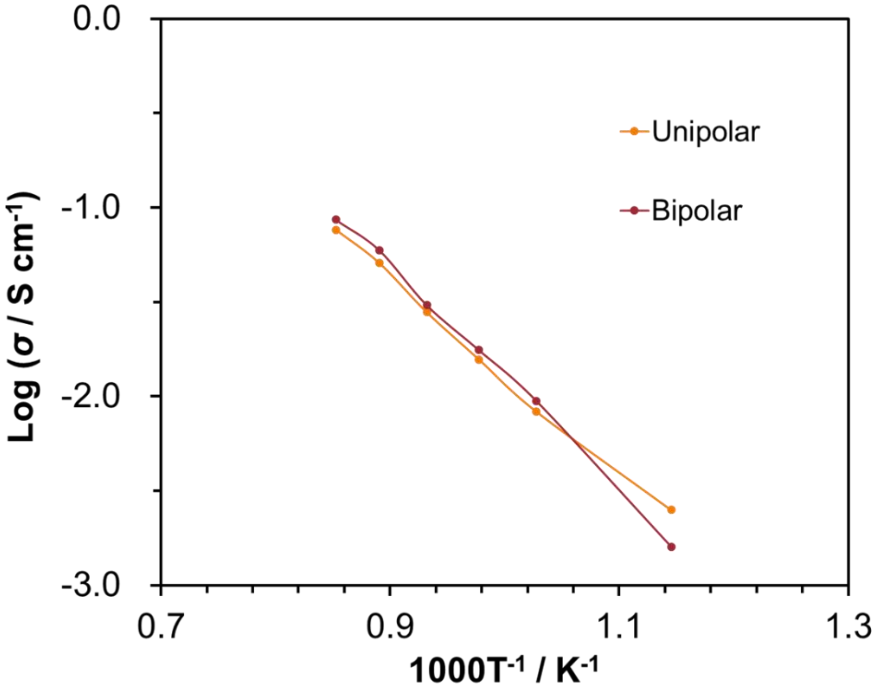

Figure S5. Temperature dependence of conductivity for $Si_{0.97}Al_{0.03}H_xP_2O_{7-\delta}$ layer measured sequentially (2 times) in unipolar mode. The conductivity was calculated based on the thickness of the layer (40 μm) rather than that of the disk (3.24 mm).

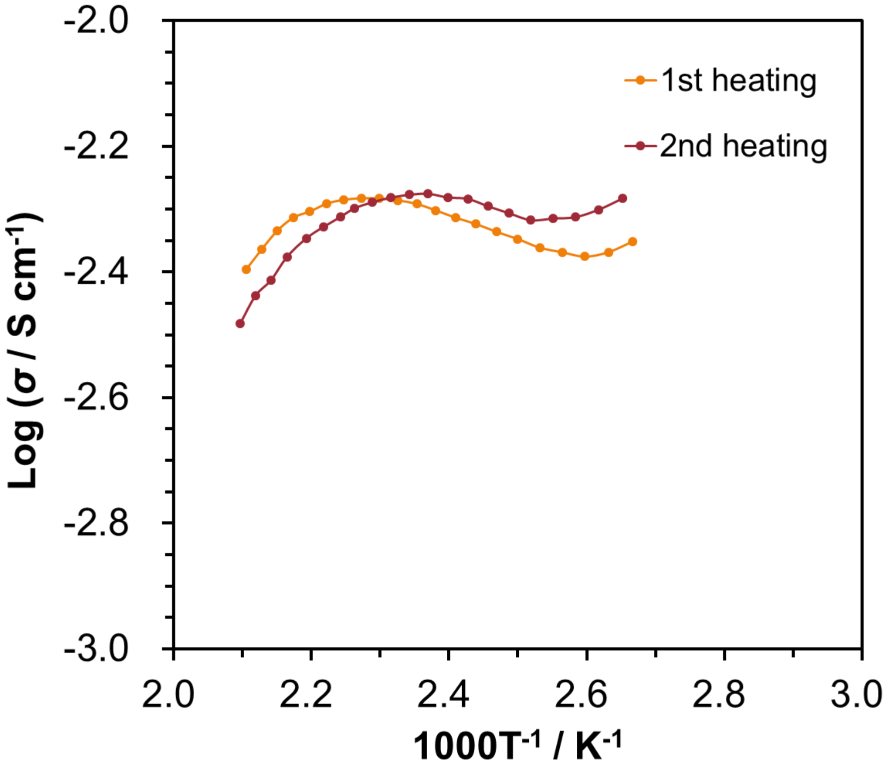

Figure S6. Transient change in OCV of the sensor upon turning the soot supply on and off.

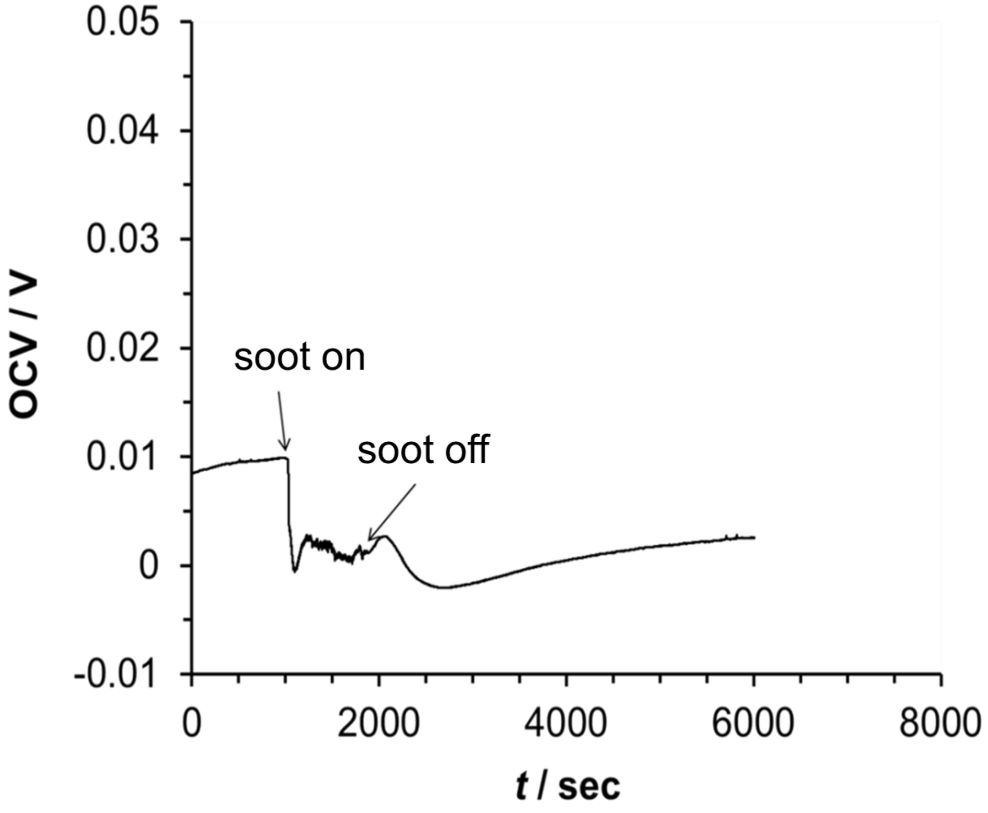

Figure S7. TG and DTA observation for soot+Pt mixed sample. These analyses were carried out under air flow at a heating rate of 5 °C min^{-1}.

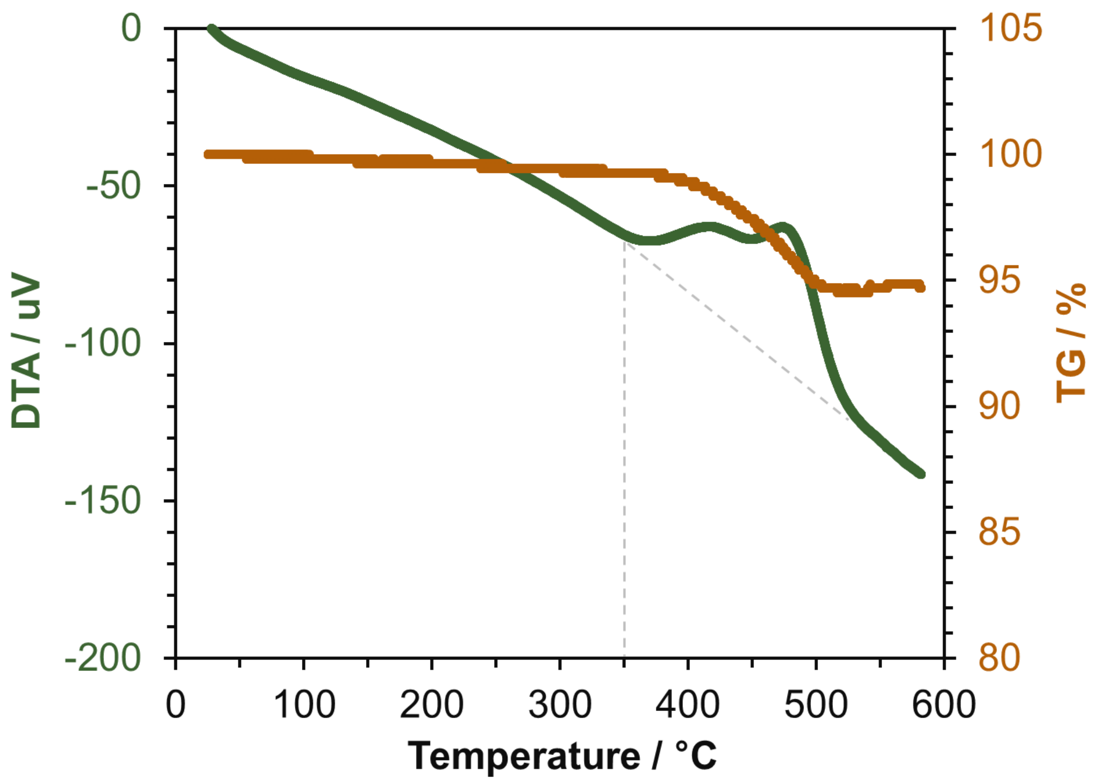

Figure S8. Raman spectra of Pt+carbon electrode at room temperature. Constant voltages were applied to the electrochemical cell under ambient atmosphere.

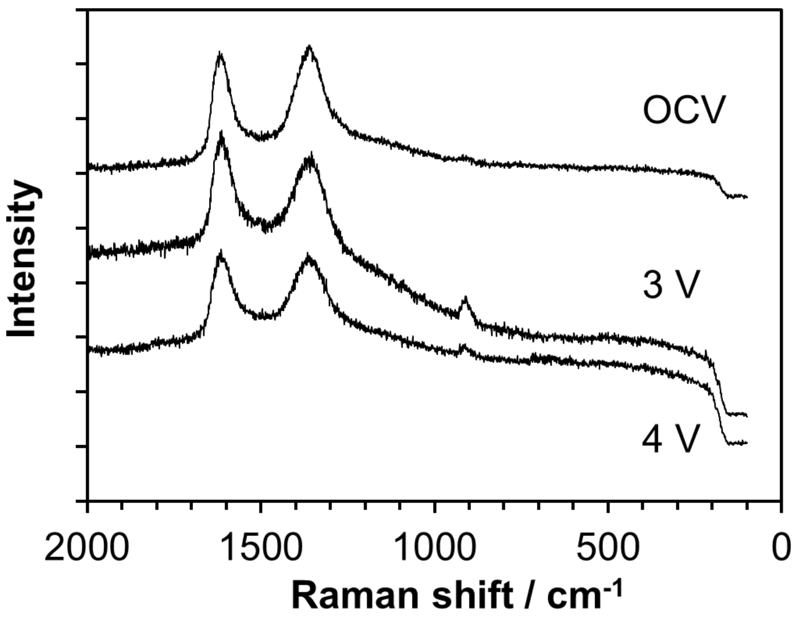

Scheme S1. Unipolar and bipolar cells for AC impedance measurements.

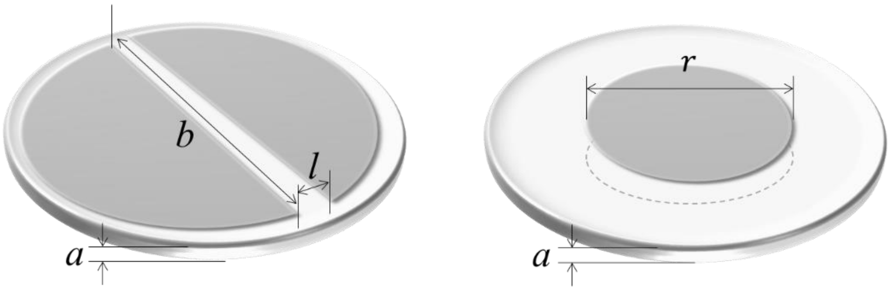

$$\sigma = \frac{l}{a \times b} \times \frac{1}{R} \qquad \sigma = \frac{a}{\pi \times r^2} \times \frac{1}{R}$$

Scheme S2. Sensor device and apparatuses employed in sensing tests.

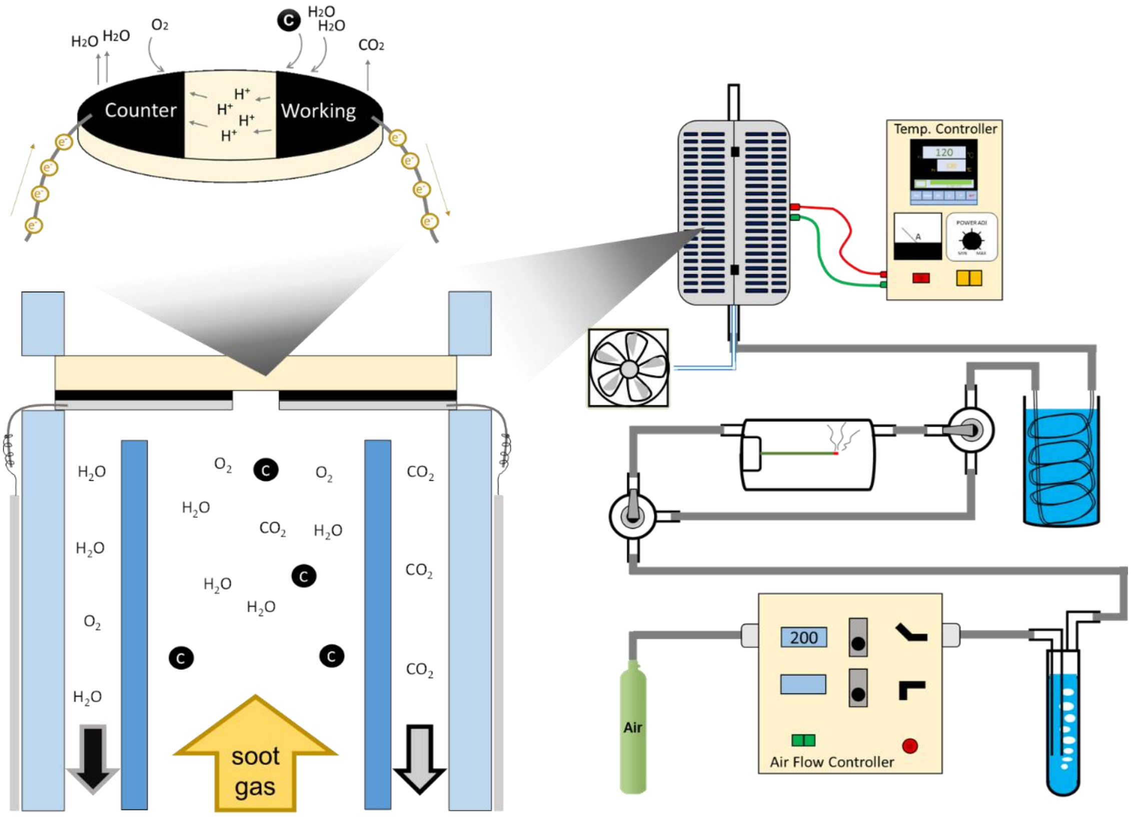

Supplement: Supplementary Information [file srep37463-s1.pdf]
